# CD44v6‐Peptide Functionalized Nanoparticles Selectively Bind to Metastatic Cancer Cells

**DOI:** 10.1002/advs.201600202

**Published:** 2016-12-20

**Authors:** Linxian Li, Mark Schmitt, Alexandra Matzke‐Ogi, Parvesh Wadhwani, Veronique Orian‐Rousseau, Pavel A. Levkin

**Affiliations:** ^1^ Institute of Toxicology and GeneticsKarlsruhe Institute of Technology76344KarlsruheGermany; ^2^ Institute of Organic ChemistryUniversity of Heidelberg69120HeidelbergGermany; ^3^ Institute of Biological Interfaces (IBG‐2)Karlsruhe Institute of Technology76344KarlsruheGermany; ^4^ Department of Applied Physical ChemistryUniversity of Heidelberg69120HeidelbergGermany

**Keywords:** antimetastatic activity, CD44v6, quantum dots, tumor targeting, v6 peptide

## Abstract

**CD44v6 peptide functionalized nanoparticles** are fabricated in a facile and controllable way to selectively bind to CD44v6 positive tumor cells with highly efficient anticancer and antimetastatic properties. The reported modular synthesis and facile preparation makes this system highly potent for developing novel multifunctional nanocarriers for therapeutic and/or diagnostic anticancer applications.

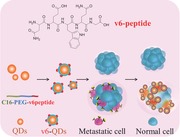

Surface functionalized nanoparticles have great potential in tumor diagnosis and targeted drug delivery. Various ligands, such as tumor targeting small molecule compounds,^1^ peptides,^2^ antibodies,^3^ and aptamers,^4^ have been immobilized on the surfaces of nanoparticles to achieve specific tumor targeting. Due to its small size and simplicity, the his‐gly‐asp (RGD) peptide is commonly used to decorate nanoparticles to achieve specific tumor targeting.[[qv: 2a]] RGD functionalized nanoparticles have exhibited efficient targeting in a variety of tumor models, i.e., pancreatic/renal orthotopic mouse tumors,^5^ murine hepatocarcinoma transplantable liver tumor (TLT),^6^ BxPC3 pancreatic cancer cells,^7^ U87MG glioblastoma,^8^ DU145 prostate tumor,^9^ and A549 lung adenocarcinoma.^10^ Other small peptides, such as CCK8,^11^ CRKRLDRNC,^12^ F3,^13^ CGKRK,^14^ and EPPT,^15^ have been used to decorate nanoparticles to achieve targeted tumor delivery. However, despite intense research on tumor targeting nanoparticles, there are very few ligands for surface modification of nanoparticles that can be conventionally synthesized while displaying highly specific and selective binding to cancer cells. There is an obvious need to raise the number and enhance the diversity of such synthetically accessible small‐molecule ligands to increase both the specificity and selectivity of tumor targeting. In addition, ligands for selective targeting of metastatic cells are even less common.

CD44 proteins correspond to a family of transmembrane glycoproteins involved in many cellular events.^16^ A variety of CD44 isoforms is generated by alternative splicing of ten variant exons that can be either completely excluded (giving rise to CD44s) or included in various combinations (giving rise to CD44 variant isoforms (CD44v)). Additional glycosylations increase the molecules' heterogeneity. The expression of several CD44v including CD44v6 is known to correlate with advanced stages of colorectal cancer, breast cancer, lung cancer, thyroid carcinoma, hepatocellular carcinoma, gall bladder carcinoma, ovarian carcinoma, and endometrial cancer.^17^ CD44v6 expression in CD44 negative Bsp73 AS pancreatic carcinoma cells conferred metastatic potential to these cells.^18^ One explanation for the participation of CD44v6 in the metastatic process of such pancreatic carcinoma cells is its involvement in Met and VEGFR‐2 signaling. Indeed, we have shown that CD44v6 acts as a coreceptor for the receptor tyrosine kinases Met and VEGFR‐2,^19^ both involved in tumor angiogenesis and metastatic spread. We identified three amino acids (aa) in the v6 sequence, which are essential for the function of CD44v6 as a coreceptor for these receptor tyrosine kinases (RTKs).^20^ We designed small peptides containing these three amino acids (termed v6‐peptides), and these peptides were able to both bind to CD44v6 expressing cells and block the function of CD44v6 protein in Met and VEGFR‐2 signaling.[[qv: 19b,20]] Experiments in mouse models for pancreatic cancer revealed that treatment with v6‐peptides inhibits tumor growth, angiogenesis and tumor metastasis in different in vivo models for pancreatic ductual adenocarcinoma.[[qv: 19b,21]] Furthermore treatment with v6‐peptides resulted in the regression of preformed metastases.^21^

In this paper, we aim to develop v6‐peptide functionalized nanoparticles that can selectively bind to metastatic cancer cells overexpressing CD44v6. We demonstrate a facile strategy to synthesize the v6‐peptide‐NHCO‐PEG‐NHCO‐palmitic amide amphiphilic polymer (v6‐PEG‐C16), and fabricate v6‐peptide functionalized nanoparticles by encapsulating hydrophobic CdTe quantum dots (CdTe‐QDs). We then demonstrate the ability of the v6‐functionalized quantum dots to selectively bind to CD44v6 positive tumor cells. In addition, we investigated the ability of the v6‐PEG to inhibit metastasis in vivo.

The first step of modification of the v6 peptide is PEGylation. To verify whether the modified v6‐peptide maintains binding affinity to CD44v6 proteins, we first PEGylated v6‐peptide and tested its efficiency in inhibiting the hepatocyte growth factor (HGF) signaling pathway. The synthesis of v6‐peptides, PEGylated v6‐peptides (v6‐PEG) as well as the amphiphilic v6‐peptide bearing the hydrophilic PEG moiety connected to a hydrophobic C16 acyl chain (v6‐PEG‐C16) is described in **Scheme** **1**. All peptide conjugates were synthesized by solid phase synthesis on Wang resin, followed by purification by preparative HPLC. PEG with average molecular weight of 2000 g mol^−1^ was used in the case of the v6‐PEG synthesis. To synthesize v6‐PEG‐C16, PEG 3000 g mol^−1^ was attached to the N‐terminus of the v6‐peptide followed by Fmoc deprotection and acylation with palmitic acid. V6‐peptide, v6‐PEG, and v6‐PEG‐C16 were purified using HPLC as previously described.^22^ The identity of the peptides was confirmed using LC‐MS and the purity of the peptides and peptide‐conjugates were found to be over 90% according to HPLC. The molecular weight was validated by matrix‐assisted laser desorption/ionization (MALDI)‐MS (**Figure** **1** A,B). Figure 1A shows the mass spectra of v6‐PEG based on PEG 2000 g mol^‐1^ and Figure 1B of v6‐PEG‐C16 based on the PEG 3000 g mol^‐1^.

**Scheme 1:**
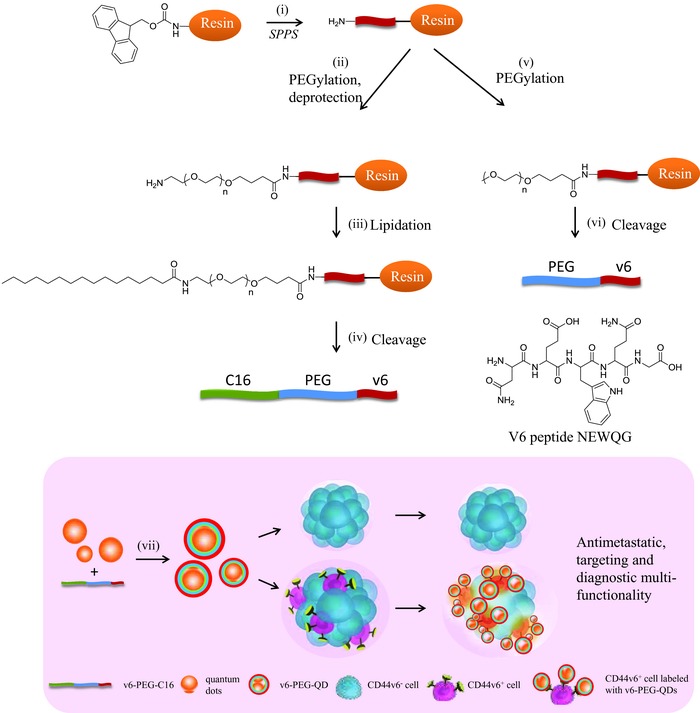
Schematic representation of the solid phase synthesis of v6‐PEG and v6‐PEG‐C16 (top) as well as nanoparticle preparation and application for targeting CD44v6^+^ cells (bottom). i) Peptide synthesis was conducted on Wang resin using standard Fmoc solid phase peptide synthesis to synthesize NEWQG, ii) Fmoc‐NH‐PEG3000‐COOH was conjugated to the free N‐terminus of the immobilized peptide; iii) After Fmoc‐deprotection, palmitic acid was conjugated to the amino terminus of the PEG using HCTU; iv) v6‐PEG‐C16 was cleaved from the resin using a mixture of TFA:H_2_O:TIS; v) MeO‐PEG2000‐COOH was conjugated to v6‐peptide by amide coupling; vi) PEG‐v6 was cleaved from the resin using a mixture of TFA:H_2_O:TIS; vii) preparation of v6‐PEG‐QDs and their selective binding to CD44v6^+^ cells. Nanoparticles were prepared by self‐assembling of hydrophobic quantum dots with amphiphilic v6‐PEG‐C16 using the lipid film method.

**Figure 1:**
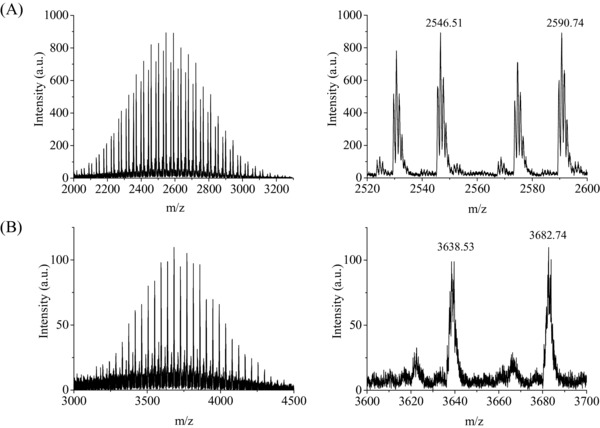
MALDI‐MS spectra of v6‐PEG based on PEG 2000 g mol^−1^ A) and v6‐PEG‐C16 based on the PEG 3000 g mol^−1^ B).

QD‐PEG‐v6 PEGylated quantum dot nanoparticles were successfully synthesized by coincubating C16‐PEG and C16‐PEG‐v6 along with QDs in chloroform, followed by evaporation of the organic solution and taking up the residue in phosphate buffer. Three different types of v6‐peptide functionalized quantum dot nanoparticles with varying v6‐peptide concentrations were prepared: PEG‐QDs (0% of v6), 10% v6‐PEG‐QD (10% of v6), and 30% v6‐PEG‐QDs (30% of v6) (see the Experimental Section).

Since QDs are stabilized with surface tetradecyl groups, we employed a self‐assembly method to encapsulate these water‐insoluble QDs in lipid nanoparticles produced from mixtures of amphiphilic PEG‐C16 and v6‐PEG‐C16.^23^ All produced nanoparticles were characterized using dynamic light scattering (DLS) (**Figure** **2**). The average hydrodynamic diameter of PEG‐QDs, 10% v6‐PEG‐QDs, and 30% v6‐PEG‐QDs were found to be 79, 106, and 132 nm, respectively (**Table** **1**). Since a single QD measures 5 nm, the DLS results indicated that about 10–20 QDs were encapsulated in a single PEG‐QD or v6‐PEG‐QD. Incorporating v6‐peptide in PEG‐QDs resulted in the particles' reduction of zeta‐potential from −14.7 mV in case of PEG‐QDs to −30.6 and −31.7 mV in the case of 10% and 30% v6‐PEG‐QDs, respectively. The decrease in zeta potential was most probably caused by the negatively charged carboxylic groups present at C‐terminus (glycine) and glutamic acid side chains in the v6‐peptide.

**Table 1:** Particle size and zeta potential of PEG‐QD, 10% v6‐PEG‐QD, and 30% v6‐PEG‐QD measured by DLS

	Mean particle size [nm]	Mean zeta potential [mV]
PEG‐QD	79 ± 16	−14 ± 5
10% v6‐PEG‐QD	106 ± 22	−31 ± 5
30% v6‐PEG‐QD	132 ± 34	−32 ± 5

**Figure 2:**
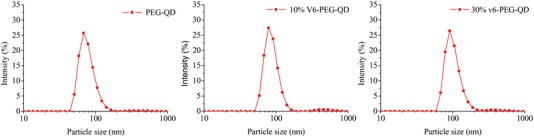
Size distribution of QD nanoparticles based on DLS with different density in v6‐peptide groups: PEG‐QDs (79 nm), 10% v6‐PEG‐QDs (106 nm), and 30% v6‐PEG‐QDs (132 nm).

V6‐peptides can efficiently block HGF‐induced hepatocyte growth factor receptor (or Met) activation and downstream signaling.^20^ Furthermore, v6‐peptide treatment of HT29 colon carcinoma cells has been shown to strongly inhibit their HGF/Met‐stimulated cell detachment and scattering. Peptide PEGylation might inhibit the peptides' binding to CD44v6 receptors, thereby resulting in a loss of biological activity. To test this hypothesis, we investigated whether the peptides were still able to block HGF‐induced Met and extracellular signal‐regulated kinase (Erk) activation after attaching a 2000 Da PEG. We treated the rat pancreatic carcinoma cell line BSp73ASs6 that highly expresses CD44v6 with v6‐PEG and tested whether this treatment impacted HGF‐induced activation of the RTK Met and its downstream target, Erk. We used v6‐peptides (A = synthesized by ourselves, B = synthesized by Bachem (Bubendorf, Switzerland)) as a positive control. We used nonPEGylated or PEGylated negative control peptides (v6 control and v6‐PEG control) where the middle three essential amino acids were replaced by alanine. As shown in **Figure** **3**, upon treating the ASs6 cells with v6‐PEG, HGF failed completely to induce Met‐phosphorylation and activate Erk signaling, whereas the v6‐PEG control displayed no effect (Figure 3A). Furthermore, HGF‐induced scattering of HT29 cells was strongly inhibited by pretreatment with v6‐PEG, but not with v6‐PEG control (Figure 3B). We then tested the v6‐PEG effect in vivo in the L3.6pl orthotopic xenograft model for pancreatic cancer.^24^ Treatment with the v6‐PEG resulted in the primary tumor's growth inhibition, similar to the v6‐peptide (**Figure** **4**A). Moreover, we detected no liver metastases in mice treated with v6‐PEG or with v6‐peptide (Figure 4B).

**Figure 3:**
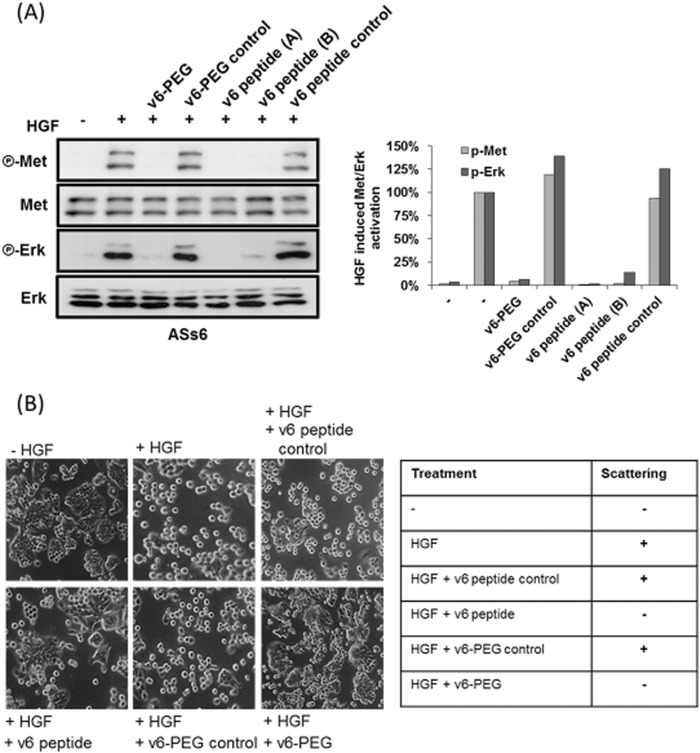
v6‐PEG efficiently blocks cell scattering and HGF/Met signaling. A) ASs6 cells treated for 30 min with 150 × 10^−9^ m of v6‐peptide (A = synthesized by ourselves, B = synthesized by Bachem, Bubendorf, Switzerland), v6‐PEG, v6 control, or v6‐PEG control were induced with HGF (10 ng mL^−1^) for 5 min. Subsequently, Met and Erk phosphorylation were determined by western‐blot analysis. p‐Met and p‐Erk levels were normalized to total Met and Erk levels, respectively. Quantification of the fold induction of activation is shown in the diagram. B) HT29 cells grown in the absence (control) or presence of HGF with or without v6‐peptide, v6‐PEG, v6 control, or v6‐PEG control were visualized by phase contrast microscopy. Magnification, 40×. The table reflects the scattering ability of HT29 cells in the absence or presence of the respective peptides.

**Figure 4:**
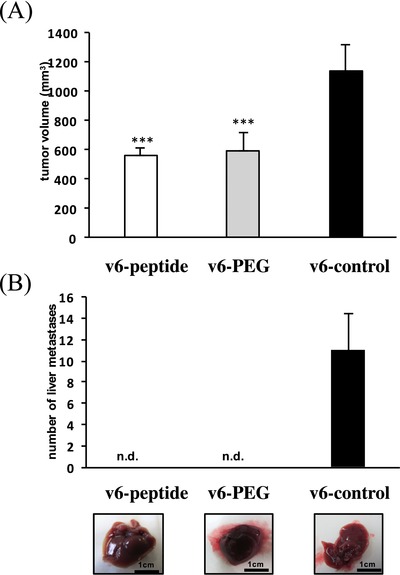
A) L3.6pl were injected orthotopically into the pancreas of male nude mice. One week later animals were injected i.p. with the v6‐peptide, the v6‐PEG, or the v6 control (20 μg). Injection was repeated three times per week. Animals were killed 3 weeks after the treatment. The quantification represents the average tumor volume of animals treated either with the v6‐peptide, the v6‐PEG or the v6 control at the end of the experiment. B) Top: Bars represent the average number of metastases. Bottom: Macroscopic liver metastases. *** *p* < 0.001, n.d.: not detected, group size *n* = 3.

Many reports have demonstrated that PEGylated proteins lose their biological activity due to the steric interference of PEG at the receptor–ligand interaction site.^25^ However, both the v6‐peptide and v6‐PEG exhibited efficient blocking in in vitro assays and in the animal model, indicating that PEGylation did not affect the v6‐peptides' functionality. As PEGylation is known to protect peptides from enzymatic degradation and to increase the half‐life of peptides in vivo,[[qv: 25c]] further investigations are necessary to evaluate whether this is also the case with v6‐peptides.

Since PEGylation did not impact the biological activities of the v6‐peptides, we further tested v6‐PEG‐QDs for their ability to specifically bind to and stain CD44v6 expressing cells. Furthermore, we analyzed whether the density of v6‐peptides on nanoparticles would influence the binding ability. We incubated CD44v6 expressing ASs6 cells (Figure S3, supporting information) or CD44v6 negative AS10 cells with PEG alone or v6‐peptide decorated QD nanoparticles, followed by confocal fluorescence microscopy (**Figure** **5**A). We quantified the percentage of QD‐stained cells (*n* = 5, Figure 5A). The PEG‐QDs alone did not stain any of the cells (whether CD44v6‐negative AS10 or CD44v6‐positive ASs6 cells (Figure 5A, panel 1; Figure 5B)). This result rules out the unspecific binding of QD nanoparticles to cells caused by the PEG layer. The 10% v6‐PEG‐QDs were found to bind to both AS10 and ASs6 cells (less than 10% cells), indicating that nanoparticles possessing low surface density of v6‐peptides interacted unspecifically with both AS10 and ASs6 cells (Figure 5A, panel 2; Figure 5B). However, 30% v6‐PEG‐QDs were found to bind to 90% ASs6 cells, whereas only 15% of the AS10 cells were stained (Figure 5A, panel 3; Figure 5B). This result demonstrates that v6‐peptides' greater surface density significantly increases the specific binding of the cargo to CD44v6‐positive ASs6 pancreatic cancer cells.

**Figure 5:**
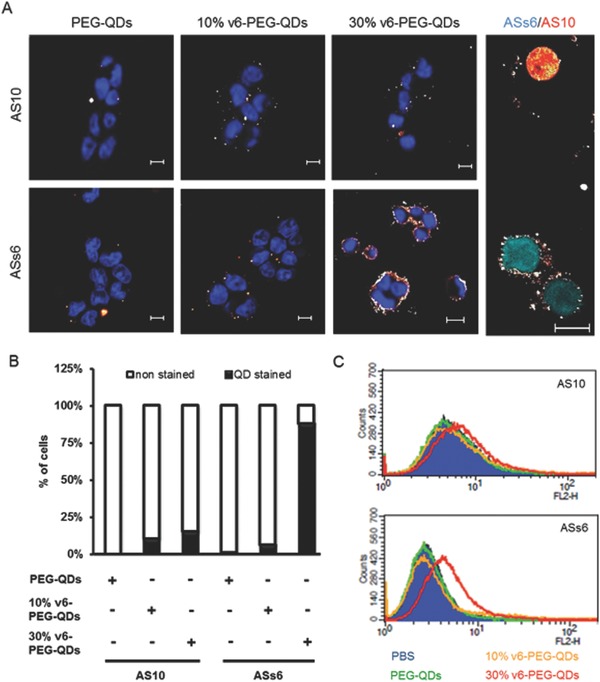
v6‐PEG‐QDs bind specifically to CD44v6 expressing tumor cells A) Panels 1–3: AS10 or ASs6 cells incubated with 100 μg mL^−1^ PEG‐QDs (control), 10% v6‐PEG‐QDs, or 30% v6‐PEG‐QDs were costained with DAPI and visualized by fluorescence microscopy. Panel 4: AS10 cells transfected with mCherry (red) and ASs6 cells transfected with CFP (cyan) were cocultured and stained with 30% v6‐PEG‐QDs. Magnification, 40×. B) Percentages of quantum dot stained cells was quantified. C) AS10 or ASs6 cells were incubated with PBS or 100 μg mL^−1^ PEG‐QDs, 10% v6‐PEG‐QDs, or 30% v6‐PEG‐QDs for 10 min and analyzed by FACS measurement.

To confirm the specific binding of v6‐peptide‐decorated nanoparticles to CD44v6‐positive cells, we transfected AS10 cells with mCherry expression vectors and ASs6 cells with CFP (cyan fluorescent protein) expression vectors, and cocultured these two cells together in one plate. We treated these cocultured cells with 30% v6‐PEG‐QDs and visualized the staining via confocal fluorescence microscopy. The result showed that the 30% v6‐PEG‐QDs only bound to CD44v6‐positive ASs6 cells (CFP labeled, Figure 5A, panel 4, cyan cells), while CD44v6‐negative AS10 cells remained virtually unstained (mCherry labeled, Figure 5A, panel 4, red cell). This result further confirms the binding specificity of the 30% v6‐PEG‐QDs toward the CD44v6‐expressing pancreatic cancer cells ASs6.

In addition to confocal fluorescence microscopy, we analyzed the cell binding activity of different v6‐peptide densities of v6‐PEG‐QDs by fluorescence activated cell sorting analysis (FACS analysis). AS10 and ASs6 cells were treated with phosphate‐buffered saline (PBS), PEG‐QDs, 10% v6‐PEG‐QDs, or 30% v6‐PEG‐QDs prior to the FACS analysis. Concurring with the fluorescence microscopic results, PEG‐QDs and 10% v6‐PEG‐QDs revealed no specific binding either to AS10 or ASs6 cells, as there was no shift in the FACS diagram compared to the nontreated PBS control (Figure 5C, green and yellow lines). On the other hand, 30% v6‐PEG‐QDs treated ASs6 cells displayed a clear shift in the FACS diagram, indicating specific binding of the high‐density v6‐peptide‐decorated QDs to the CD44v6‐expressing cancer cells (Figure 5C, red line). In contrast to this, we observed just a minor shift in the AS10 cells treated with the 30% v6‐PEG‐QDs (Figure 5C, red line). This confirms that the binding of 30% v6‐PEG‐QDs to cells is dependent on the CD44v6 expression level. These results confirm that v6‐peptide coupled to nanocarriers can be used to distinguish between cancer CD44v6^+^ and CD44v6^−^ cells. Furthermore, we used v6‐PEG‐QDs to stain CD44v6 directly on tumor tissue. For this purpose, we isolated primary tumors derived from a syngeneic rat model of pancreatic cancer.^21^ In this model CD44v6 positive ASs6 cells were subcutaneously injected in the flank of the animals. After four weeks the tumors were isolated and tumor sections were stained either with PEG‐QDs (control), v6‐PEG‐QDs or the ratCD44v6 specific antibody 1.1 ASML (Figure 5).[[qv: 18a]] Our results demonstrate that the v6‐PEG‐QDs stain CD44v6‐positive tumor tissues as also observed with a CD44v6 specific antibody (Figure S1, Supporting Information). No staining was seen with PEG‐QDs that were used as a control, demonstrating that the binding of the QDs to the tumor tissue is mediated by the coupled v6‐peptide.

Hence, v6‐PEG‐QDs might eventually be used as a powerful noninvasive theranostic tool both to treat and visualize primary tumors, metastases and metastatic pancreatic cancer cells expressing CD44v6. Further, analysis of the binding capability to CD44v6 expressing cells of other tumor types, e.g., colorectal cancer or glioblastoma should be done to reveal whether v6‐PEG‐QDs can be used more generally to detect specific (more aggressive or metastatic) subpopulations of cancer cells in other cancers as well.

In addition to the imaging advantages, v6‐peptide coupled quantum dots can serve as a platform for a combined anticancer therapy. By blocking the coreceptor function of CD44v6 for c‐Met and VEGF‐R, it is possible to inhibit protumorigenic events such as angiogenesis, tumor cell migration, and metastasis; while by coupling conventional anticancer drugs such as gemcitabine or fluororacil to the nanocarriers, the synergistic anticancer effect can be achieved. Remarkably, v6‐peptide coupled quantum dots have a cytotoxic effect specific to CD44v6 expressing pancreatic cancer cells (ASs6). We performed cell viability tests on AS10 and ASs6 cells treated with PEG‐QDs and v6‐PEG‐QDs for 48 h. At 50 μg mL^−1^ only 50% of CD44v6 expressing ASs6 cells were viable whereas PEG‐QDs treated ASs6 cells were not affected at this concentrations (Figure S2, Supporting Information). Furthermore, no cytotoxic effect of PEG‐QDs or v6‐PEG‐QDs was seen on CD44v6 negative AS10 cells (Figure S2, Supporting Information). These results suggest that the cytotoxic effect of the v6‐PEG‐QDs to ASs6 cells is dependent on the binding of the nanoparticles to CD44v6 expressing cells and might be mediated by a cellular uptake of the quantum dots.

Thus, v6‐peptide coupled quantum dots could be used to deliver cytotoxic drugs specifically to CD44v6‐expressing cancer cells without harming normal dividing cells, which would make conventional anticancer drugs more efficient and reduce their side effects. As CD44v6 is furthermore expressed in the cancer stem cells of several common cancers (e.g., colorectal cancer)^26^ and those associated with high mortality (e.g., pancreatic cancer^27^ and glioblastoma^28^) v6‐peptide coupled quantum dots might provide a platform for combined cancer treatments targeting cancer stem cells within a variety of lethal cancers.

CD44v6 expression often correlates with advanced stages of cancer and goes hand in hand with poor prognosis in various cancer types. Many malignant cancer cells, including cancer stem cells, overexpress CD44v6 on their surface, suggesting CD44v6 as a marker for several types of cancer. In this paper, we demonstrate that the functionalization of nanoparticles with a small 5‐amino acid peptide (Asn‐Glu‐Trp‐Gln‐Gly), v6‐peptide, endows nanoparticles with the ability to specifically bind cancer cells overexpressing CD44v6 receptor. The v6‐peptide was synthesized by solid phase synthesis and covalently linked to a hydrophobic C16 chain via a PEG linker to form the v6‐PEG‐C16 amphiphilic conjugate. Hydrophobic quantum dots were incorporated into v6‐PEG‐C16 lipid nanoparticles produced by the lipid film method. The addition of PEG‐C16 amphiphiles into this system enabled us to fine‐tune the density of surface v6‐peptides and reduce nonspecific binding of the particles to cells. Our results support the hypothesis that the multivalency or higher density of surface v6‐peptides is an important parameter for specific tumor targeting and essential to increasing binding specificity. In a separate experiment, we showed that apart from specific binding to CD44v6‐overexpressing cancer cells, the in vivo application of PEGylated v6‐peptide completely blocked metastasis formation and reduced the original tumor volume in a mice animal model. Such an antimetastatic effect is a result of the selective binding of v6‐peptide to CD44v6‐expressing tumor cells. To our knowledge, there is no other simple small molecule system possessing both a specific targeting ability as well as efficient anticancer and antimetastatic activities. Such dual functionality combined with the modular synthesis and facile preparation of nanoparticles decorated with the v6‐peptide makes this system highly potent for improving the existing and for developing novel multifunctional nanocarriers for therapeutic and/or diagnostic anticancer applications.

## Experimental Section

*Materials*: Human adenocarcinoma cell line HT29, a gift of A. Zweibaum (Institut National de la Sante et de la Récherche Medicale, France) was grown in Dulbecco's modified eagle's medium (DMEM) (Invitrogen, Karlsruhe, Germany) supplemented with 10% fetal calf serum (FCS) (PAA, Cölbe, Germany). Rat pancreatic carcinoma cells BSp73AS10 (AS10) and its transfectant BSp73ASs6 (ASs6) were grown in Roswell Park Memorial Institute (RPMI) (Invitrogen) plus 10% FCS. Hydrophobic CdTe quantum dots were ordered from PlasmaChem GmbH (Germany, catalogue number PL‐QDN‐550) functionalized with tetradecylamine (emission at 550 ± 5 nm, particles molar weight is 32 000 Da, size 2.6 nm). Palmitic acid (99%) and poly(ethylene glycol) methyl ether (MW 2000 g mol^−1^) were obtained from Sigma‐Aldrich (Germany). All amino acids were purchased either from Novabiochem (Schwalbach, Germany) or Iris Biotech GmbH (Marktredwitz, Germany). The α‐methoxy‐omega‐carboxylic acid poly(ethylene glycol) (MeO‐PEG‐COOH, MW 750 Da and 2000 Da), α‐(9‐fluorenylmethyloxycarbonyl)amino‐omega‐carboxy poly(ethylene glycol) (Fmoc‐NH‐PEG‐COOH, MW 3000 Da), 2‐(1H‐benzotriazol‐1‐yl)‐1,1,3,3‐tetramethyluronoium hexafluorphosphate (HBTU), 2‐(6‐chloro‐1H‐benzotriazol‐1‐yl)‐ 1,1,3,3‐tetramethyluronoium hexafluorphosphate (HCTU), 1‐hydroxybenzotriazole (HOBt), trifluoroacetic acid (TFA), triisopropylsilane (TIS), *N*‐methyl‐2‐pyrrolidone (NMP), and *N*,*N*‐diisopropylethylamine (DIEA) were purchased from Iris Biotech GmbH (Marktredwitz, Germany). Peptide were synthesized by following Fmoc‐based solid phase peptide synthesis^22^ and characterized using analytical HPLC (Agilent, Germany) coupled to electron spray mass spectrometer (Bruker Daltoniks, Bremen Germany).

*Instrumentation*: Dynamic light scattering was performed on a Malvern Zetasizer Nano ZS (Malvern, Germany). CdTe quantum dots nanoparticles were diluted with MilliQ water to a concentration of 0.1 mg mL^−1^. 750 μL nanoparticle solution was added in a cuvette, and particle size (hydrodynamic diameter) and surface charge (zeta potential) were measured.

Quantum dot nanoparticles were characterized by scanning electron microscopy (SEM) using the LEO 1530 Gemini scanning electron microscope (Zeiss, Germany, Institute of Nanotechnology, KIT). Samples for SEM were prepared by resuspending the particles in MilliQ water at a concentration of 0.1 mg mL^−1^. One drop of the resulting dispersion was placed onto a silicon wafer and dried in air. The particles were then sputter coated with gold for 20 s using a Cressington 108 auto sputter coater (Watford, UK) before measuring SEM.

Matrix‐assisted laser desorption/ionization time‐of‐flight (MALDI‐TOF) mass spectrometry was performed on a 4800 MALDI‐TOF/TOF mass spectrometer (Applied Biosystems, Framingham, MA). Peak lists were generated using Data Explorer Software 4.0 (Applied Biosystems). 2,5‐Dihydroxybenzoic acid (10 mg in 1 mL tetrahydrofuran containing 0.1% trifluoroacetic acid) was used as a matrix for PEGylated peptides and v6‐PEG‐C16. 10 μL PEGylated peptides and peptide‐PEG‐C16 (1 mg in 1 mL MilliQ) were mixed with 10 μL matrix, and 1 μL mixed solution was spotted on a stainless steel MALDI plate.

Peptide synthesis was performed on an automated peptide synthesizer (model syroII from Multisyntech, Witten, Germany) and the peptides were purified by a preparative HPLC‐system (Jasco, Tokyo, Japan). Crude and purified products were characterized by a LC‐MS (μTOF LC‐MS from Bruker Daltonics‐Bremen, Germany). Preparative HPLC was performed on a reversed phase C18 column (vydac, 10 mm × 240 mm) at 35 °C using a flow rate of 7 mL min^−1^ fitted with a diode array detector. Peptides and polymer‐peptide conjugates were separated using acetonitrile/water gradients supplemented with 0.1% of TFA.

*Peptide Synthesis and Polymer Conjugation*: Peptide synthesis, PEGylation, and palmitic acid conjugation were performed using standard Fmoc solid phase peptide synthesis protocol on wang resin.^22^ Fmoc deprotection was performed with 20% (vol) piperidine in NMP. Coupling was performed using a mixture of Fmoc‐amino acid (or MeO‐PEG‐COOH, Fmoc‐NH‐PEG‐COOH, palmitic acid): HOBt:HBTU:DIEA (4:4:3.9:8 mol/mol) in dimethylformamide (DMF). Peptides were cleaved from the solid support using a mixture of TFA:H_2_O:TIS (93.5:2.5:4 v/v). The cleavage cocktail was removed under a gentle stream of N_2_, followed by precipitation of the crude peptide using diethyl ether.

*Synthesis of PEG‐C16*: 500 mg palmitic acid was dissolved in 30 mL of CH_2_Cl_2_ and treated with 312 μL *N*,*N*′‐diisopropylcarbodiimide, 250 mg 4‐dimethylaminopyridine, and MeO‐PEG‐OH (4 g, average MW 2000 g mol^−1^) by stirring under nitrogen gas for 16 h at room temperature. The reaction solution was diluted with 100 mL cold diethyl ether and then cooled on ice. The white precipitate was filtered off and washed with sodium hydroxide solution (pH 9) to obtain PEG‐C16.

*Preparation of Nanoparticles*: V6‐peptide functionalized quantum dot nanoparticles were prepared as follows: (a) PEG‐QDs were prepared by mixing 300 μL PEG2000‐palmitic ester (PEG‐C16, 4 mg mL^−1^ in chloroform) and 30 μL QDs (10 mg mL^−1^ in chloroform) in 4 mL vials. After vortexing for 20 s, the mixed solution was kept in an open vial overnight to slowly evaporate chloroform. 300 μL PBS buffer (pH 7.4) were added to the 4 mL vial, and vortexed for 30 s to form PEGylated quantum dot nanoparticles (PEG‐QDs). (b) 10% v6‐PEG‐QDs were prepared by mixing 270 μL PEG‐C16 (4 mg mL^−1^ in chloroform), 120 μL v6‐PEG‐C16 (0.5 mg mL^−1^ in chloroform), and 30 μL QD (10 mg mL^−1^ in chloroform) in 4 mL vials. After vortexing for 20 s, the mixed solution was evaporated overnight. 300 μL PBS buffer (pH 7.4) were added to the vial, and vortexed for 30 s to form v6‐peptide conjugated (10%) PEGylated quantum dot nanoparticles (v6‐PEG‐QDs). (c) 30% v6‐PEG‐QDs were prepared by mixing 210 μL PEG‐V16 (4 mg mL^−1^ in chloroform), 360 μL v6‐PEG‐C16 (0.5 mg mL^−1^ in chloroform), and 30 μL QD (10 mg mL^−1^ in chloroform) in 4 mL vials. After vortexing for 20 s, the mixed solution was left open overnight to evaporate chloroform. 300 μL PBS buffer (pH 7.4) was added to the vial and vortexed for 30 s to form nanoparticles. Since a single QD measures 5 nm, the DLS results indicated that about 10–20 QDs were encapsulated in a single PEG‐QD or v6‐PEG‐QD.

*Western Blotting and Activation of Met and Erk*: ASs6 cells, serum starved for 24 h, were induced with HGF (10 ng mL^−1^) at 37 °C for 5 min. Where indicated, the cells were treated with PEGylated or nonPEGylated peptides (150 × 10^−9^ m) before induction at 37 °C for 10 min. Cells were washed with PBS and lysed in boiling sodium dodecyl sulfate (SDS) buffer containing 100 × 10^−3^ m dithiothreitol (DTT). Subsequently, lysates were subjected to western blot analysis using antibodies against phosporylated Mek and Erk (Cell signaling). The Mek and Erk loading controls were performed on the same blot, stripped (62.5 × 10^−3^ m Tris pH 6.8, 2% SDS, 0,8% DTT) and probed with the Mek and Erk antibodies. Blots were stained using the enhanced chemiluminescence system (Thermo Scientific, Dreieich, Germany).

*Scattering Assay*: Scattering of HT29 cells was determined in 24‐well plates. First, 4 × 10^5^ cells were seeded at 37 °C; the cells were either left untreated or 48 h later treated with HGF (50 ng mL^−1^) for 24 h or with PEGylated or nonPEGylated peptides (100 ng mL^−1^) prior to HGF induction for 30 min.

*Confocal Fluorescence Microscopy*: 1 × 10^4^ AS10 and ASs6 cells per well were seeded on 20 mm glass coverslips (Menzel, Braunschweig, Germany) and incubated for 24 h at 37 °C. The cells were washed with ice‐cold PBS, fixed with methanol for 15 min at −20 °C, and again washed three times with PBS. Afterward, cells were incubated with the indicated modified quantum dots for 1 h at room temperature in the dark. Subsequently, cells were stained with 4,6‐diamidino‐2‐phenylindole dihydrochloride (DAPI) in PBS for 30 min at room temperature, washed three times with PBS, and mounted with fluorescence mounting medium (Dako, Glostrup, Denmark). Fluorescence images were taken with a Leica SPE laser scanning confocal microscope with a 63× objective.

*Fluorescence Activated Cell Sorting*: ASs6 and AS10 cells cultured in 10 cm petri dishes were harvested at 80% confluency, with 5 × 10^−3^ m EDTA/PBS. Cells were washed three times with 3% FCS in PBS. Afterward, cells were incubated with 100 ng mL^−1^ quantum dots in 3% FCS/PBS for 10 min. Then, cells were washed three times with 3% FCS in PBS, resuspended in PBS without FCS, and subjected to FACS measurement.

*Cell Viability Assay*: ASs6 or AS10 cells grown in a 96‐well tissue culture plate (0,5 × 10^4^/well) were incubated with the indicated concentrations of PEG‐ and v6‐PEG‐QDs for 48 h. For measuring cell viabilty cells were incubated with 10 μL well^−1^ Cell Proliferation Reagent WST‐1 for 2 h and mixed for 1 min on a shaker. The absorption at 420 nm was determined with an ELISA reader EXL 808 (Biotek). Culture medium and WST‐1 reagent in empty wells were used as blank controls.

*Animals*: Male athymic nude mice (NCI‐nu) were purchased from Harlan (Roßdorf, Germany). BDX rats were bred in house. Animals were housed and maintained under specific pathogen‐free conditions in facilities approved by the Regierungspräsidium Karlsruhe. All animals were handled according to German regulations for animal experimentation. Experiments were authorized by the Regierungspräsidium (35‐9185.817G‐192/10 and 35‐9185.817G‐106/09).

*Orthotopic Implantation of Tumor Cells*: L3.6pl cells (passage 28) were suspended in Hank's balanced salt solution (Invitrogen) after trypsinization. The cells were injected into the pancreas of NCI‐nu as previously described.^24^ Mice were injected i.p. 7 d after tumor cell implantation with the v6‐peptide, the v6‐PEG or the control peptide (20 μg) where the three central amino acids were substituted with alanines (NAAAG). The injection was repeated three times per week for 21 d.

*Immunofluorescence Stainings of Tumor Tissue*: The syngeneic rat pancreatic cancer model is described previously.^21^ Briefly, 1 × 10^6^ BSp73ASs6 cells were injected subcutaneously into the right posterior flank of BDX rats. Tumors developed for 4 weeks. At the end of the experiment, primary tumors were isolated. The tissues were snapfrozen in liquid nitrogen and cut into 4–6 μm thick sections that were mounted on gelatin‐coated glass slides. The sections were fixed in acetone 1:1 for 10–15 min at 4 °C. The sections were washed in PBS and blocked at room temperature for 1 h with 5% FCS in PBS. For immunohistochemistry, tissues were incubated with the CD44v6 specific antibody 1.1ASML[[qv: 18a]] (5 μg mL^−1^) in blocking solution over night at 4 °C. After washing three times with PBS, sections were incubated with 2nd Alexa488‐antibodies (Invitrogen) in blocking solution for 2 h at room temperature. Second antibodies samples were used as control. For quantum dot stainings, the tissues were incubated either with PEG‐QDs or 30% v6‐PEG‐QDs (50 μg mL^−1^ in blocking solution) for 1 h at room temperature. Subsequently to the stainings with antibodies or QDs, cells were stained with DAPI in PBS for 30 min at room temperature, washed three times with PBS, and mounted with fluorescence mounting medium (Dako, Glostrup, Denmark). Fluorescence images were taken with a Leica SPE laser scanning confocal microscope.

*Fluorescence‐Activated Cell Sorting (FACS)*: Expression of CD44v6 in ASs6 cells was measured by FACS analysis. Cells were seeded at a concentration of 5 × 10^5^ cells in 5 cm plates. After 48 h cells were detached from the plates using PBS supplemented with 5 × 10^−3^ m ethylendiamine tetraacetate (EDTA) and resuspended in PBS containing 3% FBS Gold. 1 × 10^6^ cells were incubated with 1.1ASML (1 μg mL^−1^) for 60 min on ice followed by three washing steps. After 30 min of incubation with antimouse fluorescein isothiocyanate (FITC)‐labeled secondary antibody on ice and additional three washing steps the cells were resuspended in PBS. The fluorescence was analyzed in a FACScan flow cytometer (Becton Dickinson, Heidelberg, Germany).

## Supporting information

As a service to our authors and readers, this journal provides supporting information supplied by the authors. Such materials are peer reviewed and may be re‐organized for online delivery, but are not copy‐edited or typeset. Technical support issues arising from supporting information (other than missing files) should be addressed to the authors.

Supplementary
