# Supplementary material for: CD44v6‐Peptide Functionalized Nanoparticles Selectively Bind to Metastatic Cancer Cells
Source: Adv Sci (Weinh). 2016 Dec 20;4(1):1600202. doi: 10.1002/advs.201600202 (PMC5238741; doi:10.1002/advs.201600202)
Supplement: Supplementary file 1 — Supplementary [file ADVS-4-0-s001.pdf]

Copyright WILEY-VCH Verlag GmbH & Co. KGaA, 69469 Weinheim, Germany, 2016.

Supporting Information

for *Adv. Sci.,* DOI: 10.1002/advs.201600202

CD44v6-Peptide Functionalized Nanoparticles Selectively Bind to Metastatic Cancer Cells

Linxian Li, Mark Schmitt, Alexandra Matzke-Ogi, Parvesh Wadhwani, Veronique Orian-Rousseau, and Pavel A. Levkin**

Supporting Information

CD44v6-peptide functionalized nanoparticles selectively bind to metastatic cancer cells

Linxian Li,[a, b, †] Mark Schmitt,[a, †] Alexandra Matzke-Ogi,[a] Parvesh Wadhwani,[c] Veronique Orian-Rousseau,[a] Pavel A. Levkin[a, d,*]*

Dr. L. Li, Dr. M. Schmitt, Dr. A. Matzke-Ogi, Prof. V. Orian-Rousseau, Dr. P. A. Levkin A
Institute of Toxicology and Genetics, Karlsruhe Institute of Technology, 76344 Karlsruhe, Germany
E-mail: veronique.orian-rousseau@kit.edu; levkin@kit.edu
Dr. L. Li
Institute of Organic Chemistry, University of Heidelberg, 69120 Heidelberg, Germany
Dr. P. Wadhwani
Institute of Biological Interfaces (IBG-2), Karlsruhe Institute of Technology, 76344 Karlsruhe, Germany
Dr. P. A. Levkin
Department of Applied Physical Chemistry, University of Heidelberg, 69120 Heidelberg, Germany

Keywords: Quantum Dots, CD44v6, Tumor Targeting, v6 Peptide, Antimetastatic Activity

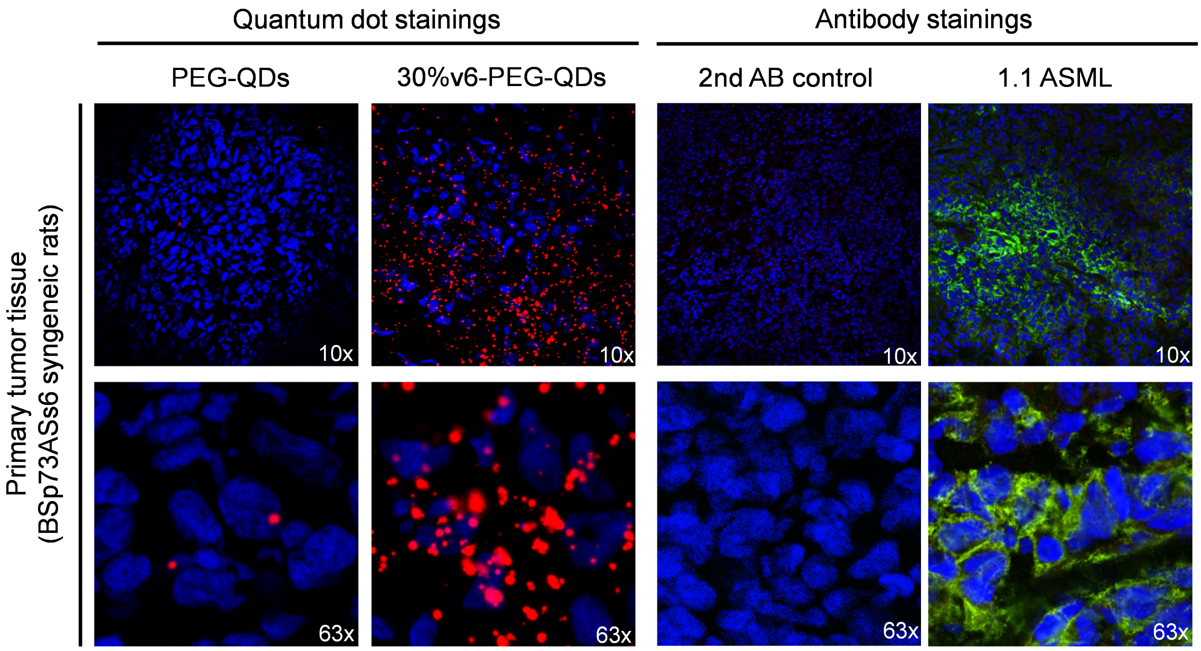

Figure S1. v6-PEG-QDs stain CD44v6 positive tumor tissues. Sections of primary tumors from BSp73ASs6 injected syngeneic rats were stained either with PEG-QDs (control; panel1), 30% v6-PEG-QDs (panel 2), 2nd antibody only (control; panel 3) or the rCD44v6 specific antibody 1.1ASML (panel 4). The tissue was counterstained with DAPI and analysed by confocal fluorescence microscopy. Picture show 10x (upper lane) and 63x magnifications (lower lane).

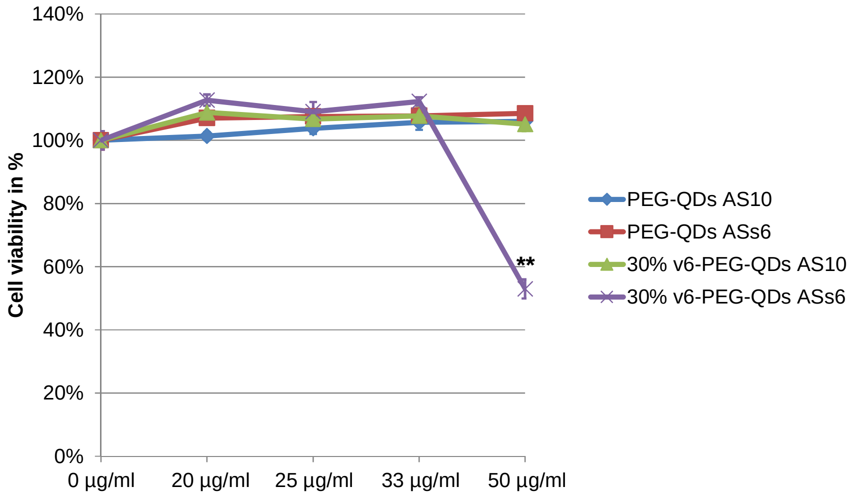

Figure S2. v6-PEG-QDs mediated cytotoxicity is specific to CD44v6 expressing pancreatic cancer cells. Ass6 and AS10 cells were incubated for 48 hours with the indicated concentrations of PEG-QDs (control) and v6-PEG-QDs and cell viability was analysed using a WST-1 assay (** P<0,01, AS10 and Ass6 cells treated withv6-PEG-QDs were compared, n=3).

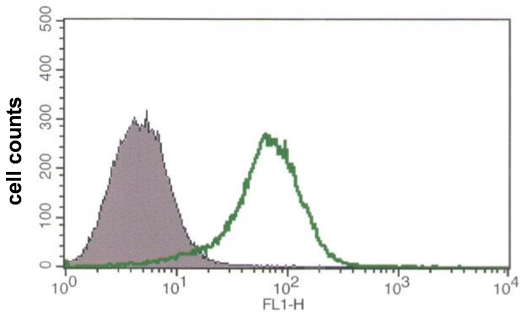

Figure S3. CD44v6 expression in ASs6 cells. Bsp73ASs6 (ASs6) cells were stained with a CD44v6 antibody (1.1ASML). All samples were analyzed in a FACScan flow cytometer.

Unstained cells are shown in grey, cells stained with 1.1ASML are shown in green. Representative pictures from one experiment are shown.
